# Kissing as a Protective Factor Against Decreased Salivary pH: Protocol for a Randomized Clinical Trial

**DOI:** 10.2196/65253

**Published:** 2025-07-17

**Authors:** Marcelo Armijos Briones, Mariuxi Aguila Gaibor, Andrea Bermúdez Velásquez

**Affiliations:** 1 Dental School Universidad Católica de Santiago de Guayaquil Guayaquil Ecuador

**Keywords:** Saliva, oral health, sugar-sweetened beverages, dental caries prevention, kissing, protective, social behavior, stress, relationship, pH, fermented drinks, randomized controlled trial

## Abstract

**Background:**

Kissing is a common social behavior that may influence physiological responses and impact oral health. Prior research has shown that affectionate behaviors like kissing can reduce stress and improve relationship satisfaction. However, the effects of kissing on salivary pH, particularly after consuming sugary or fermented beverages, have not been thoroughly investigated.

**Objective:**

This study aims to determine whether kissing accelerates salivary pH recovery following the consumption of sugary or fermented beverages.

**Methods:**

A randomized controlled trial will be conducted with 60 couples aged 18-30 years. Participants will be divided into 3 groups: a control group (no kissing), an experimental group where one partner consumes a beverage, and another experimental group where both partners consume a beverage. The study will measure salivary pH at baseline, after beverage consumption, and following a 40-second kiss or control period, with measurements taken every 5 minutes until the pH returns to neutral levels. The data will be analyzed using SPSS software (IBM Corporation). ANOVA will be used to compare salivary pH recovery between groups, and if assumptions of normality or sphericity are not met, alternative models such as generalized linear models or nonparametric tests will be considered.

**Results:**

The study is ongoing, with participant recruitment already initiated and preliminary data collection in progress. The study was funded by the Catholic University of Santiago de Guayaquil from June 2024 to June 2025. Ethics approval was obtained in June 2023. The results are expected to be published in the last quarter of 2025.

**Conclusions:**

This study will provide insights into the relationship between kissing, salivary pH, and oral health, potentially offering new strategies for the prevention of dental caries. The results may challenge existing assumptions about the role of kissing in oral health.

**Trial Registration:**

ClinicalTrials.gov NCT06501729; https://clinicaltrials.gov/study/NCT06501729

**International Registered Report Identifier (IRRID):**

DERR1-10.2196/65253

## Introduction

Dental caries remains one of the most prevalent chronic diseases worldwide, particularly among adolescents and young adults [1]. One key factor contributing to its development is the frequent consumption of sugar-sweetened and fermented beverages, which can significantly lower salivary pH and compromise the protective capacity of saliva [2,3]. The acidic environment created by these beverages facilitates enamel demineralization and disrupts oral microbiome balance, increasing susceptibility to caries.

Studies have shown that after consuming cariogenic food or drink, the salivary pH decreases significantly within the first 5 minutes and may take up to 60 minutes to return to baseline levels. Another study indicates that the intraoral pH drops after drinking acidic beverages and that it takes approximately 20-30 minutes for the saliva to neutralize any residual acid [4], [5]. These findings highlight the prolonged acidic exposure that the enamel undergoes following the intake of such beverages, emphasizing the importance of strategies to expedite pH normalization [6].

The pH of body fluids is a measure of the hydrogen ion concentration within them, reflecting their acidity or alkalinity. The normal pH range for extracellular fluid (ECF) is tightly regulated between 7.35 and 7.45. The pH of intracellular fluid is slightly lower, ranging from 7.1 to 7.2. It is essential to maintain these pH levels within narrow limits to ensure proper enzymatic and metabolic processes [7]. Interestingly, despite the vast difference in concentration between hydrogen ions and other ions, such as sodium ions, the body’s regulatory mechanisms are adept at maintaining the pH within the necessary range for life, which is between 6.8 and 7.8 in the ECF. Any deviation from the normal pH range can lead to significant physiological disturbances because pH influences the structure and function of proteins and affects various physiological processes. In summary, the pH of body fluids is a critical physiological parameter that must be maintained within a narrow range. The body uses various mechanisms to regulate this balance, ensuring optimal functioning of cellular and systemic processes [8].

Several studies have demonstrated that common beverages, such as carbonated soft drinks, fruit-based juices, and coffee, cause an immediate and notable drop in salivary pH after ingestion. For instance, the salivary pH dropped from a baseline of 7.18 to 5.65 immediately after consuming a cola-based soft drink, and from 7.05 to 6.08 following the intake of a mango-flavored juice, both requiring up to 15 minutes to return to baseline values. Coffee, although less acidic than sodas, also reduced salivary pH from 7.05 to 6.10. These changes, even if transient, can become harmful when acidic drinks are consumed repeatedly throughout the day [9].

Milk-based beverages, even when sweetened, tend to produce only minimal changes in salivary pH. A study reported a slight decrease from 7.07 to 6.71 after ingestion, with near-immediate normalization [10]. These findings emphasize the importance of beverage selection in maintaining oral pH stability.

The methodology used to assess these changes typically involves pH meters with digital glass electrodes, which allow precise and repeatable measurements. Several studies evaluating the effect of acidic drinks on saliva have used this approach to monitor real-time fluctuations in pH across multiple time points [11]. In this context, the ability of saliva to restore pH levels after an acidic challenge is essential for maintaining enamel integrity. Although artificial methods, such as chewing sugar-free gum or using alkaline rinses, have been proposed to accelerate salivary recovery, little attention has been given to natural behaviors that may play a similar role. One such behavior is kissing. During a French kiss, a substantial volume of saliva is exchanged between partners, and it is plausible that this could introduce buffering agents and bacteria from one individual to another, potentially supporting faster pH normalization.

While the physiological benefits of kissing—such as reduced cortisol levels, improved mood, and lowered blood pressure—have been well documented [12,13], no studies to date have explored whether kissing may contribute to the recovery of salivary pH following acidic challenges. This represents a novel and unexplored area of research within oral health and preventive dentistry.

Given that kissing involves significant salivary exchange, it is hypothesized that this behavior may introduce buffering agents from one individual’s saliva to another, potentially accelerating the neutralization of oral acidity following the consumption of acidic beverages. To test this hypothesis, we designed a randomized controlled trial with three conditions: a control group (no kissing), an experimental group where only one person drinks and then kisses, and another group where both individuals drink and then kiss. Additionally, three types of acidic beverages commonly consumed in the local context, Coca-Cola, Cifrut, and nonalcoholic beer will be used to assess whether the effect of kissing on salivary pH recovery is consistent across different sources of oral acidification. This design allows for both behavioral and beverage-specific comparisons to better understand the potential protective role of kissing. The objective of this study is to determine whether the act of kissing facilitates a faster recovery of salivary pH compared to the natural physiological restoration process following the intake of acidic beverages.

## Methods

### Ethical Considerations

A trained member of the research team will clearly and concisely explain the study’s objectives, procedures, potential risks, and benefits to the participants. Each participant will receive a copy of the informed consent form, which has been approved by the Council on Ethics in Human Research from the Instituto Superior Universitario “Portoviejo” (CEISH-ITSUP). This document provides comprehensive details about the study and outlines participants’ rights, including their ability to withdraw at any time without penalty. Participants will be given sufficient time to review the form and ask any relevant questions. Both members of each couple must provide their consent to participate in the study.

The investigator responsible will document the informed consent process, ensuring that each participant’s signature and the date of consent are properly recorded. Personal information, including participants’ names, contact details, and unique identification codes, will be collected during enrollment and stored separately from the study data. Participants will not receive any compensation for their participation.

This study was reviewed and approved by the CEISH-ITSUP (681428096; Multimedia Appendix 1). The study was registered in Clinical Trials (NCT06501729; Multimedia Appendix 2). The study protocol was structured according to the SPIRIT (Standard Protocol Items: Recommendations for Interventional Trials) guidelines (Multimedia Appendix 3).

### Study Design

The protocol will be carried out using a parallel 3-arm randomized controlled design. It will evaluate whether the act of kissing on the mouth between 2 people can cause the pH of saliva to increase after consuming sugary or fermented beverages. The initial pH will be obtained from participants who meet the inclusion criteria. After recording the initial pH, participants will be given 200 mL of one of the beverages selected for the study (Coca-Cola, Cifrut, or nonalcoholic beer). After ingesting the beverage, the participants will be asked to drool into a test tube to measure the pH of the saliva using a pH meter. Based on the literature, it is expected that the pH will decrease. After this measurement, the experimental groups will be asked to kiss their partners, and the control group will not do so. The pH of the saliva will be measured for a third time 5 minutes after ingesting the beverage to record the changes. This will be done repeatedly, every 5 minutes, until the pH of the saliva reaches a neutral level (6.8-7.2).

The difference between the experimental groups is that, in one, only one person from the kissing couple will have a sugary or fermented beverage, and in the other, both individuals will consume the sugary or fermented beverage. Each week, a different beverage will be tested; that is, in week 1, Coca-Cola will be used, in week 2 artificial fruit juice, and in week 3 nonalcoholic beer.

**Figure 1:**
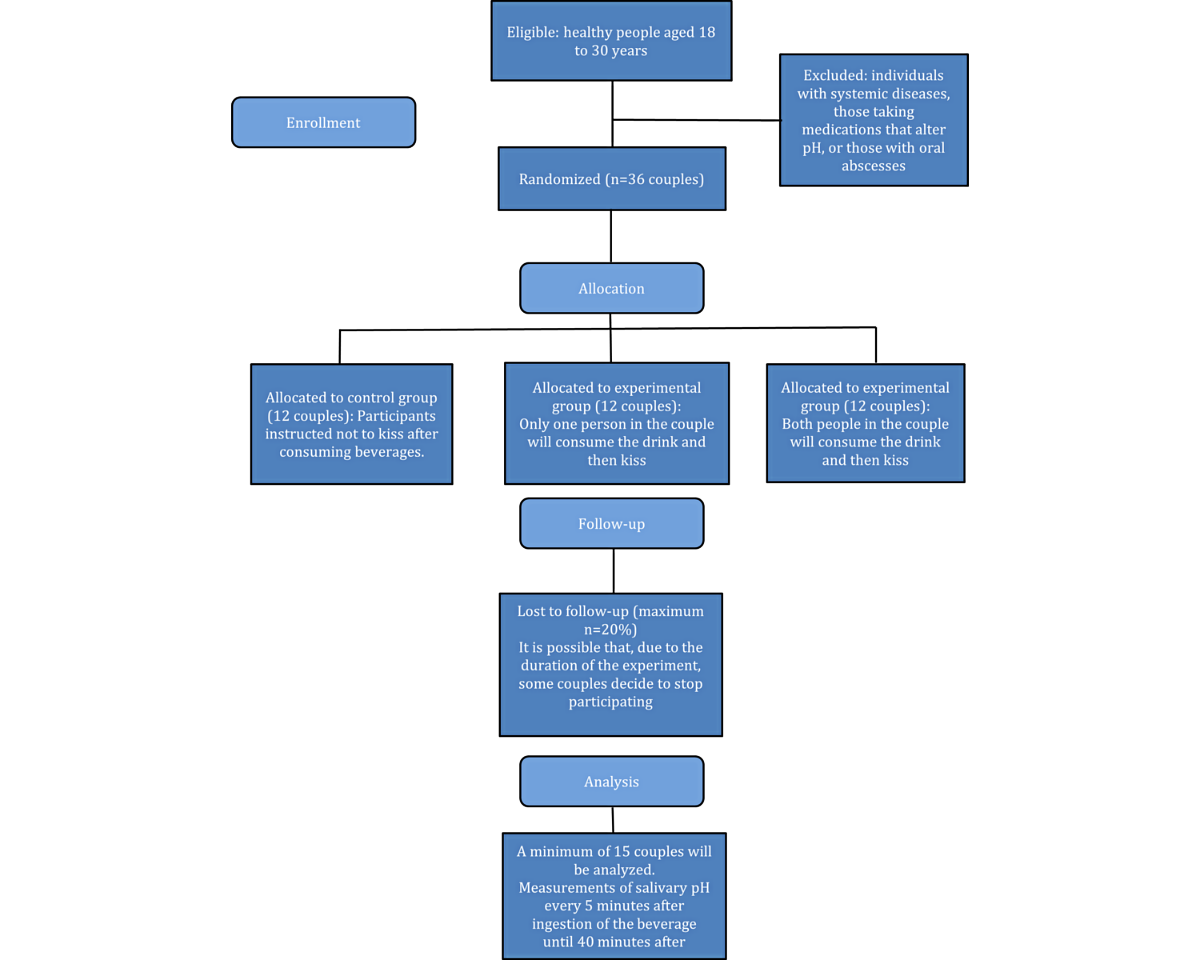
CONSORT (Consolidated Standards of Reporting Trials) flow diagram of the study design outlining the selection and randomization of couples participating in the study. In total, 36 couples will be required to try 3 different beverages.

This randomized controlled trial will be conducted according to the CONSORT (Consolidated Standards of Reporting Trials) checklist and guidelines, as outlined in Figure 1 [14].

### Eligibility Criteria

Some physiological and pathological conditions could alter the pH of saliva, which could bias the results of this study. Therefore, the aim is to select couples aged between 18 and 30 years, the age at which the probability of having chronic degenerative diseases is lower. In addition, all volunteers who enroll will undergo an oral examination to determine their oral health, and those with abscesses, extensive carious cavities (level 5 or 6 according to the International Caries Detection and Assessment System), periodontal disease, or other alterations that may modify the salivary pH will be excluded. Furthermore, participants must not undergo medical treatment that requires them to consume medications that change the pH of their saliva.

These criteria were selected to minimize confounding factors that could alter salivary pH, such as systemic diseases, abscesses, or the influence of medications. The age range of 18-30 years was chosen to reduce the likelihood of undiagnosed chronic systemic diseases, as no specific tests will be conducted to confirm their absence; participant responses will be accepted as valid. Additionally, requiring participants to already have a romantic partner ensures that the study is conducted ethically and avoids potential discomfort from pairing participants with strangers.

The recruitment process will involve an open call via social media platforms targeting university students, although being a student is not an inclusion criterion. Flyers with a QR code and a registration link will direct interested individuals to a Google Form where they will provide initial data. Eligible participants will be contacted to schedule an oral health assessment and complete a health questionnaire to confirm compliance with the inclusion criteria.

### Sample Size

To select the participating couples, a call will be made to students of the Universidad Católica de Santiago de Guayaquil and members of the general public within the established age range. To participate in the study, participants must agree, together with their partner, to be part of the research and sign the informed consent form. The couples will be divided into three groups: 1 control group and 2 experimental groups. However, all participants must register with their partners because the group assignment will not be revealed until the day of saliva sample collection. The sex of the participants will be balanced among the groups, ensuring equal representation of men and women. Additionally, all participants must meet the inclusion criteria detailed in this document.

The sample size was calculated using the G*Power statistical software (version 3.1.9.7, Heinrich-Heine-University). Data from a pilot study by Uma et al [11] were used to estimate the necessary sample size. This study provided the following values for the calculation: control group mean 7.10, SD 0.18 and experimental group mean 6.86, SD 0.18. Based on these parameters, the effect size for ANOVA was calculated to be 0.76, which corresponds to a large effect size.

Using the parameters α=.05, power=0.95, and 3 groups, the G*Power software indicated that a minimum of 30 participating couples (10 per group) is required. To account for potential dropouts, an additional 20% (n=6) will be recruited, resulting in a total of 36 participating couples (12 couples per group). This calculation ensures sufficient statistical power while maintaining logistical feasibility.

### Randomization

Randomization will be carried out by an external researcher to whom lists of coded pairs will be given. The researcher will use the randomized website to create 3 lists, one for each group. In this way, each couple will be assigned to 3 different groups. The control group will include participants who will not engage in the kissing intervention. This group serves as a baseline for evaluating the natural recovery process of salivary pH after consuming acidic beverages. The first experimental group will have one partner consume the acidic beverage, and then the couple will engage in a 40-second kiss. This allows evaluation of the effect of kissing when only one partner has an acidic pH alteration. The second experimental group will have both partners consume the acidic beverage before engaging in the 40-second kiss. This setup examines whether mutual exposure to acidic conditions influences the efficacy of kissing in restoring salivary pH. In summary, the groups will be as follows:

1. Control: one partner drinks, no kiss
2. Experimental 1: one partner drinks, both kiss
3. Experimental 2: both partners drink, both kiss

### Intervention

The act of kissing will be an intervention that we try to associate with an increase in salivary pH after its decrease. To do so, the experimental groups will kiss with their partner after ingesting one of the beverages, followed by a series of pH measurements. First, the baseline pH of the participants’ unstimulated saliva will be taken, and then they will be given 200 mL of the beverage to be tested that week. After verifying that the beverage was completely ingested by the participants, they will wait 1 minute before providing a new saliva sample to verify the decrease in pH. After recording the second measurement, the participants in the experimental groups will be asked to kiss with their partner for 40 seconds. Participants will be instructed to make this kiss “passionate.” After that time, participants will wait 4 minutes and 20 seconds (remaining time to complete 5 minutes), and a saliva sample will be taken again to record the pH. Except for the act of kissing, the same will be done with the control group, with a saliva sample taken every 5 minutes for the next 40 minutes, which is when physiological re-establishment of salivary pH is expected.

A pH meter measures the hydrogen ion activity in a solution, which is indicative of its acidity or basicity. The device typically uses a glass electrode as a pH sensor, which interacts with hydrogen ions in the solution to produce an electrical potential that can be translated into a pH value. This value is displayed on a readout, often a liquid crystal display, which may also show temperature readings owing to the influence of temperature on pH measurements.

The saliva sample for pH measurement will not be stimulated because the increase in fluid alters the pH; thus, a reduced sample is expected to be obtained. For this reason, a thin electrode will be used so that it can enter the bottom of the test tube and capture the measurement. For this purpose, the Mettler Toledo InLab Micro pH sensor with the following technical characteristics was chosen:

- Micro combined pH electrode with a glass body and S7 threaded head
- Measuring range: pH 0-14
- Temperature zone: 0°C-80°C
- Union type: ceramic
- Shaft material: glass
- Sensor type: combined electrode
- Shaft length: 60 mm
- Shaft diameter: 3 mm
- Reference system: ARGENTHAL with Ag^+^ ion trap
- Reference electrolyte: 3 mol/L KCl
- Glass membrane: U
- Membrane resistance (25C): <600 M

### Study Outcomes

The first outcome will be the time required for salivary pH to return to baseline after being lowered by kissing a person who has not consumed a beverage. To determinate this, salivary pH will be measured every 5 minutes using a calibrated pH meter. The second outcome will be the time required for salivary pH to return to baseline after 2 people kiss, both of whom will have consumed a beverage that lowers pH. Finally, the average salivary pH of each group will be compared at each 5-minute interval.

### Data Analysis

Data analysis will be performed using SPSS Statistics software (version 21.0; IBM Corporation). Data will be presented as descriptive statistics and examined for missing values, outliers, normality, and homogeneity. To compare descriptive variables between groups, the *t* test will be used for quantitative variables and the chi-square test for categorical variables. Kolmogorov-Smirnov and Levene tests will be applied to assess normality and homogeneity of variances, especially for salivary pH measurements. Comparisons of mean salivary pH values between the 3 groups will be conducted using repeated-measures ANOVA if assumptions are met. When normality or sphericity assumptions are violated, alternative methods such as the Friedman test or generalized linear models with interaction terms and covariates (eg, type of beverage and time point) will be considered. Additionally, within-group changes in salivary pH over time will be analyzed using paired-sample tests or their nonparametric equivalents.

## Results

The study was funded by the Catholic University of Santiago de Guayaquil from June 2024 to June 2025. Ethics approval was obtained by the CEISH-ITSUP (681428096) in June 2023. The call for participants began in early May 2025. As of the date of this submission, 30 couples have expressed interest in participating. Recruitment is ongoing, and data collection is expected to continue over the coming months. As of July 2025, we have collected data for Coca-Cola and Cifrut. Sample collection is extensive, and getting participants to continue throughout the study is proving difficult. The results are expected to be published in the last quarter of 2025.

## Discussion

### Principal Findings

This study proposes an innovative hypothesis that the act of kissing may accelerate the recovery of salivary pH following the consumption of acidic beverages. By facilitating salivary exchange between individuals, kissing may help neutralize the acidic environment created by sugary or fermented drinks. The findings of this study could have implications for how natural interpersonal behaviors contribute to maintaining oral pH homeostasis.

The experimental design allows for the comparison of 3 groups to evaluate whether the buffering capacity of one partner’s saliva can affect the oral environment of the other. This investigation seeks to determine whether the salivary pH in participants who engage in kissing returns to neutral more quickly than in those who do not kiss. This may serve as evidence of an additional, natural protective mechanism against acidogenic dietary habits—one that involves behavioral factors rather than pharmacological or mechanical intervention.

### Comparison With Prior Work

Previous studies have confirmed that the consumption of sugary or acidic beverages leads to a rapid decline in salivary pH, often reaching values below the critical threshold for enamel demineralization. These drinks can prolong an acidic oral environment for up to 40 minutes or more, depending on individual salivary flow and buffering capacity [15,16]. Although several approaches have been proposed to counteract this effect, such as dairy product intake, chewing sugar-free gum, or using mouth rinses, no previous studies have evaluated whether natural behaviors like kissing could offer similar benefits.

Other research has explored nonconventional approaches. For instance, the effect of fermented yogurt on the oral cavity demonstrated positive outcomes for maintaining salivary pH [16], and pineapple juice fermented with probiotics showed a limited but measurable influence on pH recovery [17]. However, these studies relied on biochemical mechanisms rather than behavioral dynamics. This study is the first to evaluate whether intimate human interaction can function similarly by transmitting buffering agents through saliva, thus supporting oral pH recovery.

### Limitations

This study has several limitations that should be acknowledged. The experimental conditions involve an arbitrary time of 40 seconds for kissing, which lacks direct precedent in the literature. Additionally, salivary pH measurements will be taken every 5 minutes, a frequency commonly used in similar protocols but still based on convention rather than robust comparative trials. The beverages used in this study—Coca-Cola, Cifrut, and nonalcoholic beer—reflect consumer preferences in Guayaquil, Ecuador, but may not be representative of other populations.

Furthermore, couples who participate may not kiss in the same way as they would in private, as some participants may feel inhibited or perform differently knowing they are part of a research setting. These behavioral variables may influence the results and are difficult to control.

Another limitation is related to sample composition. Most participants will be university students from middle-income urban backgrounds. This restricts the generalizability of the findings to the broader population, especially those with different sociodemographic profiles or baseline oral health conditions.

### Implications

Should this study demonstrate a measurable benefit of kissing in restoring salivary pH, it would introduce a novel behavioral approach to oral health maintenance. While this is not intended to replace traditional preventive methods, such as brushing, flossing, and dietary control, it may serve as a complementary insight into how natural human interactions influence oral physiology.

This concept may also have implications for health education strategies. For example, oral health promotion campaigns, particularly those targeting adolescents and young adults, could benefit from including accessible, behaviorally relevant messages. Digital platforms and mobile health applications that disseminate oral hygiene habits might consider integrating insights from this study to make content more relatable and engaging [18,19].

### Future Research

Future studies could build upon these findings by exploring the effects of varying kiss durations, frequency, and intensity on salivary pH recovery. It would also be valuable to assess whether long-term behavioral patterns involving intimate salivary exchange can influence the oral microbiome and its association with dental caries. Further interdisciplinary research combining microbiology, behavioral science, and preventive dentistry is encouraged. Such efforts could help establish whether kissing may be a meaningful component in the broader framework of natural strategies for oral health preservation.

### Conclusions

The objective of this study is to investigate the impact of kissing on salivary pH and its potential function in preventing dental caries. The findings of this study will provide valuable insights into the relationship between kissing and oral health. Previous research has suggested that salivary pH can affect the development of dental caries, but the role of kissing in this process has not been extensively studied. This study is crucial because it could lead to a better understanding of the factors that contribute to oral health and the prevention of dental caries. The results of this study will be compared to those of previous research on the relationship between salivary pH and dental caries. The findings of this study may contradict existing beliefs regarding the role of kissing in oral health. The study will be supported by relevant literature and data on saliva pH and dental caries. This study will contribute to the field of oral health research and provide valuable information for the development of preventive measures against dental caries.

Despite the limitations described in our research, the methodology that is intended to be applied, the times of the measurements, and the choice of beverages are supported by other studies that will allow our results to be replicable, verifiable, and comparable with other research. Furthermore, if our hypothesis can be verified, we will have a new preventive measure against the decrease in salivary pH and its consequent risk of dental caries, which is as common and free as kisses between 2 people who are in a relationship.

## Appendix

Multimedia Appendix 1 Ethics committee approval.

Multimedia Appendix 2 Clinical trial registration.

Multimedia Appendix 3 SPIRIT (Standard Protocol Items: Recommendations for Interventional Trials) checklist, study timeline, and model consent form.

## Abbreviations

CEISH-ITSUP: Council on Ethics in Human Research from Instituto Superior Tecnológico de Portoviejo
CONSORT: Consolidated Standards of Reporting Trials
ECF: extracellular fluid
SPIRIT: Standard Protocol Items: Recommendations for Interventional Trials

## Data Availability

The measurements will be recorded in a web-based Excel sheet and in a physical form for the researchers’ backup. The data will be available after completing the collection process in a web-based database available to the general public with the respective anonymization of the study participants.
