# Supplementary material for: Kissing as a Protective Factor Against Decreased Salivary pH: Protocol for a Randomized Clinical Trial
Source: JMIR Res Protoc. 2025 Jul 17;14:e65253. doi: 10.2196/65253 (PMC12314465; doi:10.2196/65253)
Supplement: Multimedia Appendix 1 [file resprot_v14i1e65253_app1.pdf]

Anexo 23. Respuesta a la Solicitud de Estudios Observacionales y de Intervención

Para: Marcelo Armijos Briones
CC:

Título del Protocolo: "Potencial efecto protector del beso bucal entre dos personas contra la disminución del pH salival"
Protocolo #: 1681428096
Versión: 1
Fecha de recepción: 13/04/2023
Código CEISH ITSUP: CEISH-ITSUP.023 - 2023

Por medio de la presente se informa la decisión del CEISH sobre su solicitud de aprobación de protocolos para el estudio Potencial efecto protector del beso bucal entre dos personas contra la disminución del pH salival" que fue recibido por el Comité de Ética de Investigación en Seres Humanos (CEISH) de ITSUP, en (13/04/2023).

**JUSTIFICACIÓN DEL INFORME**		
SIN OBSERVACIÓN		
**OBSERVACIONES**		
SIN OBSERVACIÓN		
**RECOMENDACIONES**		
SIN OBSERVACIÓN		
**DECISIÓN**		
1	Exento de aprobación	
2	Aprobación definitiva	X
	Aprobación condicionada a modificaciones, aclaraciones o informaciones complementarias	
4	No aprobación del protocolo de investigación	

**Código CEISH-ITSUP**	CEISH-ITSUP.023 - 2023

Cabe señalar que, la aprobación de su solicitud lo obliga a presentar informes sobre su investigación y la elaboración y presentación del informe final del mismo a este comité. Además, en caso de realizar enmiendas al estudio aprobado, estas requieren la evaluación y aprobación del CEISH – ITSUP para su implementación.

Cualquier pregunta, correspondencia y formas, envíelas al correo electrónico del CEISH ITSUP: comité.etica@itsup.edu.ec, o llame al 0985059632.

Firmado electrónicamente por:
JOHANNA MABEL SANCHEZ RODRIGUEZ

Cordialmente, (Nombre)
Presidente del Comité de Ética de Investigación en Seres Humanos (CEISH)

(19/06/2023)
Fecha de Correspondencia
