# Supplementary material for: Kissing as a Protective Factor Against Decreased Salivary pH: Protocol for a Randomized Clinical Trial
Source: JMIR Res Protoc. 2025 Jul 17;14:e65253. doi: 10.2196/65253 (PMC12314465; doi:10.2196/65253)
Supplement: Multimedia Appendix 2 [file resprot_v14i1e65253_app2.pdf]

ClinicalTrials.gov PRS DRAFT Receipt (Working Version)
Last Update: 07/09/2024 10:17

ClinicalTrials.gov ID: NCT06501729

Study Identification

Unique Protocol ID: 598

Brief Title: Kissing as a Protective Factor Against Acidic pH in Saliva

Official Title: Potential Effect of Oral Kissing Between Two People Against the Decrease in pH

Secondary IDs:

Study Status

Record Verification: July 2024

Overall Status: Not yet recruiting

Study Start: July 30, 2024 [Anticipated]

Primary Completion: August 30, 2024 [Anticipated]

Study Completion: November 30, 2024 [Anticipated]

Sponsor/Collaborators

Sponsor: Universidad Catolica Santiago de Guayaquil

Responsible Party: Principal Investigator
Investigator: Fernando Armijos Briones [fbriones]
Official Title: Teaching researcher
Affiliation: Universidad Catolica Santiago de Guayaquil

Collaborators:

Oversight

U.S. FDA-regulated Drug: No

U.S. FDA-regulated Device: No

U.S. FDA IND/IDE: No

Human Subjects Review: Board Status: Approved
Approval Number: 1681428096
Board Name: CEISH ITSUP
Board Affiliation: CEISH ITSUP
Phone: +593 985059632
Email: comité.etica@itsup.edu.ec
Address:

García Moreno Street and América Avenue. Portoviejo, Equador.

Data Monitoring: Yes

FDA Regulated Intervention: No

Study Description

Brief Summary: The pH of saliva can be acidic or alkaline; When it is acidic, the tooth enamel is demineralized, that is, weakened. Additionally, bacteria in the mouth can increase and cause infections such as cavities. This occurs when we consume sugary or fermented drinks, such as soda or beer.

The intention of the research team is to give some of these drinks to the study participants and verify the decrease in salivary pH. Next, a group of people will be asked to kiss their partner on the mouth, and then the pH will be measured again to see if it increases, that is, if it stops being acidic faster than in the group that did not kiss.

The aim is to demonstrate that kissing on the mouth between two people can protect teeth from cavities by rapidly increasing the pH of saliva after it has decreased to become acidic.

Detailed Description: The goal of this intervention study will be to determine if oral kissing between two people can increase salivary pH more quickly after drinking artificial juices, soft drinks, or (non-alcoholic) beers. It will also be studied whether the pH restoration time is faster when only one person kisses one of the named drinks or when both people do it. The main questions that are intended to be answered are the following:

- Is the act of kissing on the mouth capable of increasing the pH of saliva more quickly than physiologically after consuming a sugary or fermented drink that has decreased it?
- Is the increase in salivary pH after an oral kiss more accelerated when only one person consumes sugary or fermented drinks than when both people do so? The investigators will measure the basal pH of all participants before providing them with one of the drinks (artificial fruit juice, soda, or non-alcoholic beer). The researchers will then give the drinks and measure the decrease in salivary pH in all groups. Control group participants will not kiss; They will only wait for the salivary pH to neutralize physiologically. The rest of the couples will be divided into two groups: the group in which only one person in the couple consumes the sugary or fermented drink, and the group in which both people in the couple do so.

Saliva pH measurements will be performed, after initiation and consumption of the drinks, every 5 minutes for up to 40 minutes. That is, until the restoration of salivary pH is ensured in all groups.

Participants:

- They will be between 18 and 30 years old to reduce the probability of consuming medications that alter salivary pH.
- They should not be taking medications that alter the pH of saliva.
- They must be in good health.
- They should not have abscesses in the oral cavity.
- They must have good oral health according to the loss proposed and the observed index.

Conditions

Conditions: Dental Caries
Tooth Demineralization

Teeth Erosion Limited to the Enamel

Keywords: Saliva
Hydrogen-Ion Concentration
dental caries

Study Design

Study Type: Interventional

Primary Purpose: Prevention

Study Phase: N/A

Interventional Study Model: Sequential Assignment
The couples in the control group will not kiss; they will only wait for the salivary pH to neutralize physiologically. The other couples will be divided into two groups: the group in which only one person in the couple consumes the sugary or fermented drink, and the group in which both people in the couple do so.

Number of Arms: 3

Masking: Single (Participant)

Allocation: Randomized

Enrollment: 45 [Anticipated]

Arms and Interventions

Arms	Assigned Interventions
No Intervention: Control<br>The couples in the control group will not kiss; they will only wait for the salivary pH to neutralize physiologically.	
Experimental: Only one person consumes the drink<br>the group in which only one person in the couple consumes the sugary or fermented drink and they kiss	Kiss<br>Participants will kiss with their partners for 40 seconds. The kiss will be on the mouth.
Experimental: Both people consume the drink<br>In this group, both people will consume the drinks and then kiss.	Kiss<br>Participants will kiss with their partners for 40 seconds. The kiss will be on the mouth.

Outcome Measures

Primary Outcome Measure:

1. salivary pH
   It is expected to measure the progressive increase in pH until reaching a neutral point, which occurs physiologically between 30 to 40 minutes, and compare the time spent by the control group with that of the experimental group. Measurements will be taken every 5 minutes from the kiss, which will last 40 seconds. In total, there will be 8 measurements, not counting the initial one for surveying the baseline. The measurement will be carried out with a calibrated pH meter. People will be asked to drool into a test tube

   [Time Frame: In a period of every 5 minutes, until reaching 40 minutes, which is the time in which physiologically the pH of the saliva is neutralized.]

Eligibility

Minimum Age: 18 Years

Maximum Age: 30 Years

Sex: All

Gender Based: No

Accepts Healthy Volunteers: Yes

Criteria: Inclusion Criteria:

- They must be in good health
- They must have good oral health according to the raised loss and observed index.
- They will be between 18 and 30 years old to reduce the probability of consuming medications that alter salivary pH.

Exclusion Criteria:

- Be taking medications that alter the pH of saliva.
- Have abscesses in the oral cavity

Contacts/Locations

Central Contact Person:

Central Contact Backup:

Study Officials: NOTE : Study Official is required by the WHO and ICMJE.

Locations: **Ecuador**
School of Dentistry of the Catholic University Santiago de Guayaquil
Guayaquil, Guayas, Ecuador, 17-11-5058
Contact: Andrea C Bermúdez, Dra. +593 985020014
andrea.bermudez@cu.ucsg.edu.ec

IPDSharing

Plan to Share IPD: Undecided
NOTE : Plan to Share IPD must be 'Yes' or 'No' to satisfy ICMJE policy ('Undecided' is not accepted).

References

Citations: Uma E, Theng KS, Yi LLH, Yun LH, Varghese E, Soe HHK. Comparison of Salivary pH Changes after Consumption of Two Sweetened Malaysian Local Drinks among Individuals with Low Caries Experience: A Pilot Study. Malays J Med Sci. 2018 Jul;25(4):100-111. doi: 10.21315/mjms2018.25.4.10. Epub 2018 Aug 30. PubMed 30914852

Links:

Available IPD/Information:
