# Supplementary material for: Kissing as a Protective Factor Against Decreased Salivary pH: Protocol for a Randomized Clinical Trial
Source: JMIR Res Protoc. 2025 Jul 17;14:e65253. doi: 10.2196/65253 (PMC12314465; doi:10.2196/65253)
Supplement: Multimedia Appendix 3 [file resprot_v14i1e65253_app3.pdf]

SPIRIT 2013 Checklist

Section/ item	No	Description
Title	1	Protocol: Kissing as a protective factor against decreased salivary pH. A randomized clinical trial.
Trial registration	2a	**Trial Registration:**<br>This trial has been registered in **ClinicalTrials.gov** under the identifier **NCT06501729**. ClinicalTrials.gov is a publicly accessible platform that adheres to international standards for trial transparency and accessibility.
	2b	**WHO Trial Registration Data Set**<br>1. **Primary Registry and Trial Identifying Number:**<br>ClinicalTrials.gov, NCT06501729.<br>2. **Date of Registration in Primary Registry:**<br>[Insert registration date]<br>3. **Secondary Identifying Numbers:**<br>Not applicable.<br>4. **Source(s) of Monetary or Material Support:**<br>Universidad Católica de Santiago de Guayaquil.<br>5. **Primary Sponsor:**<br>Universidad Católica de Santiago de Guayaquil.<br>6. **Contact for Public Queries:**<br>Name: Dr. Marcelo Armijos Briones<br>Email: [Insert email]<br>Phone: [Insert phone number]<br>7. **Contact for Scientific Queries:**<br>Same as above.<br>8. **Public Title:**<br>Potential Protective Effect of Kissing on Salivary pH Recovery After Acidic Beverage Consumption.<br>9. **Scientific Title:**<br>Potential Protective Effect of Buccal Kissing Between Two People on the Reduction of Salivary pH.<br>10. **Countries of Recruitment:**<br>Ecuador.<br>11. **Health Condition(s) or Problem(s) Studied:**<br>Salivary pH restoration, prevention of dental caries.<br>12. **Intervention(s):**<br>o **Experimental group:** Kissing intervention after acidic beverage consumption.<br>o **Control group:** No kissing after acidic beverage consumption.<br>13. **Key Inclusion and Exclusion Criteria:**<br>o **Inclusion:** Participants aged 18-28, good oral health, availability of a partner.<br>o **Exclusion:** Systemic diseases affecting salivary pH, medications altering salivary pH, severe oral pathologies.<br>14. **Study Type:**<br>Interventional.<br>15. **Date of First Enrollment:**<br>march 2025<br>16. **Target Sample Size:**<br>45 couples (15 per group).<br>17. **Recruitment Status:**<br>Not yet recruiting

		18. **Primary Outcome(s):**<br>Time required for salivary pH to return to neutral after intervention.<br>19. **Key Secondary Outcomes:**<br>Salivary pH levels at specific time intervals.
Protocol version	3	Second version: 04/01/2025
Funding	4	**1. Financial Support:**<br>The study is fully funded by the **Universidad Católica de Santiago de Guayaquil**, which provides financial resources for the following:<br>o Laboratory equipment, including pH meters and calibration solutions.<br>o Consumables, such as sterile Eppendorf tubes and cleaning materials.<br>o Administrative costs related to participant recruitment and data management.<br>**2. Material Support:**<br>o The **Odontology Clinics of the Universidad Católica de Santiago de Guayaquil** will serve as the primary site for participant interviews, oral health assessments, and saliva sample collection.<br>o The university also provides access to private rooms equipped with dividers to ensure participant privacy during the experimental intervention (kissing).<br>**3. Other Support:**<br>o **Human Resources:** The research team consists of faculty members and trained staff from the university, who voluntarily contribute their time and expertise.<br>o **Ethical Oversight:** The project has received ethical approval from the **Ethics Committee of the Instituto Superior Universitario "Portoviejo" (ITSUP)**, ensuring adherence to ethical guidelines throughout the study.<br>o **Technical Assistance:** Randomization for the study is managed remotely by **Dr. Gabriela Zambrano**, an external collaborator, to ensure impartiality.<br>The funding and resources provided by the Universidad Católica de Santiago de Guayaquil ensure the independence of the study, with no external sponsors or commercial interests involved.
Roles and responsibilities	5a	**Names, affiliations, and roles of protocol contributor**s.<br>ABV; professor and researcher at the Faculty of Health Sciences of the Catholic University of Santiago de Guayaquil, in the Dentistry program. She reviewed the research project and analyzed the final version sent to the research department of the Catholic University of Santiago de Guayaquil. She will be in charge of managing the physical spaces where the samples will be taken and the oral cavity of future participants will be examined.
	5b	**Name and contact information for the trial sponsor.**<br>Dra. Nancy Villegas is the coordinator of the Research Center for the Faculty of Health Sciences and represents the university as a sponsor of this research. Her email address is nancy.villegas@cu.ucsg.edu.ec
	5c	**Role of study sponsor and funders.**<br>The institution sponsoring this research, including the coordinator of the Research Center, has no influence on the versions of the protocol or on the decision of what is sent to the journal for publication. They do not participate in any way in the collection or analysis of data.

	5d	**Composition, roles, and responsibilities of the coordinating center.**<br>In Ecuador, these types of clinical trials, where no medications or medical devices are used, are considered by law as intervention studies. As such, data monitoring equipment is not required, nor is the acquisition of life insurance for the research participants.
**Introduction**		
Background and rationale	6a	**Description of Research Question and Justification**<br>The primary research question for this study is: **"Can the act of kissing accelerate the recovery of salivary pH after consuming acidic beverages compared to the natural recovery process?"**<br>**Rationale for the Study:**<br>This study is motivated by the increasing prevalence of dental caries worldwide, largely influenced by frequent exposure to dietary acids, which lower salivary pH and demineralize enamel. Previous studies have explored various methods to restore salivary pH, such as the use of mouth rinses, chewing gum, and dietary interventions. However, no research has yet evaluated the potential protective effect of kissing—a natural and common behavior—on salivary pH recovery.<br>**Summary of Relevant Studies:**<br>1. **Acidic Beverage Impact on Salivary pH:** Studies have shown that beverages like sodas and citrus-based drinks significantly reduce salivary pH, which can persist for up to 40 minutes, creating a window of susceptibility for enamel erosion and caries development.<br>2. **Kissing and Salivary Exchange:** Limited literature suggests that kissing facilitates salivary exchange, which may dilute acidic components and introduce buffering agents from one partner's saliva, potentially aiding in faster pH recovery.<br>3. **Gaps in Current Knowledge:** While previous research has focused on artificial interventions, the natural interaction between two individuals during a kiss has not been studied, leaving a gap in understanding its potential health benefits.<br>This study aims to address this gap by systematically evaluating the effect of kissing on salivary pH recovery, with the goal of identifying a novel, accessible, and natural protective behavior against acidic challenges to oral health.
	6b	**Explanation for Choice of Comparators**<br>The study includes the following groups for comparison:<br>1. **Control Group:** Participants who will not engage in the kissing intervention. This group serves as a baseline for evaluating the natural recovery process of salivary pH after consuming acidic beverages.<br>2. **Experimental Group 1:** One partner consumes the acidic beverage, and the couple engages in a 2-minute kiss. This allows evaluation of the effect of kissing when only one partner has an acidic pH alteration.<br>3. **Experimental Group 2:** Both partners consume the acidic beverage before engaging in the 2-minute kiss. This setup examines whether mutual exposure to acidic conditions influences the efficacy of kissing in restoring salivary pH.<br>**Justification for Comparators:**<br>These groups were chosen to isolate the effects of kissing on salivary pH recovery and to assess the variability in outcomes when both partners have altered pH versus only one. The inclusion of a control group ensures that

		the results of the kissing intervention can be compared to the natural recovery process, providing robust insights into its potential benefits.
Objectives	7	Specifically, this proposal aims to determine whether the act of kissing on the mouth between two people can neutralize the pH of the saliva in the mouth when it is acidic.
Trial design	8	The study will be a randomized controlled trial with **parallel groups**.<br>Participants will be randomly assigned to one of three groups:<br>o **Control group:** Participants who do not kiss after consuming an acidic beverage.<br>o **Experimental group 1:** One member of the couple consumes an acidic beverage, and both partners kiss for 40 seconds.<br>o **Experimental group 2:** Both members of the couple consume an acidic beverage and kiss for 40 seconds.<br>The results will be used to evaluate whether the act of kissing restores salivary pH more quickly than the time it takes for the body to do so physiologically. This design allows for clear comparisons of the effects of the different conditions, with an appropriate control group.
**Methods: Participants, interventions, and outcomes**		
Study setting	9	The study will be conducted at the dental clinics of the **Universidad Católica de Santiago de Guayaquil**. These facilities provide a controlled and sanitized environment, adhering to strict biosecurity protocols. Data collection will take place two days per week, during which the space will be reserved exclusively for the study.<br>The following arrangements have been made to ensure participant comfort and privacy:<br>o **Oral Health Assessments:** Dental chairs within the clinic will be used to evaluate the oral health of participants prior to the intervention.<br>o **Privacy During Interventions:** Private spaces separated by screens (biombos) will be set up to allow participants to kiss in a comfortable and discreet environment.<br>o **Saliva Sample Collection:** Saliva samples will be collected and pH measurements will be performed in the same private setting immediately following the intervention.<br>This controlled environment is designed to ensure participant safety, facilitate standardized data collection, and maintain the integrity of the study.
Eligibility criteria	10	**Inclusion Criteria:**<br>Participants must meet the following criteria to be eligible for the study:<br>1. Be between **18 and 28 years old**.<br>2. Have good oral health, defined as a **CPO index ≤ 3**, according to the criteria of the World Health Organization.<br>3. Be in a romantic relationship with a partner willing to participate in the study.<br>4. Have sufficient availability to attend all study appointments.<br>**Exclusion Criteria:**<br>Participants will be excluded from the study if they meet any of the following conditions:<br>1. Presence of systemic diseases that could alter salivary pH (e.g., diabetes, autoimmune disorders).<br>2. Use of medications that may affect salivary pH, including:<br>o **Antidepressants (tricyclics)**: e.g., amitriptyline.<br>o **Antihistamines**: e.g., diphenhydramine.<br>o **Beta blockers**: e.g., propranolol.<br>o **Diuretics**: e.g., hydrochlorothiazide.

		○ **Anticholinergics**: e.g., atropine.<br>3. Presence of oral abscesses or other severe dental conditions.<br>**Justification:**<br>These criteria were selected to minimize confounding factors that could alter salivary pH, such as systemic diseases, abscesses, or the influence of medications. The age range of **18 to 28 years** was chosen to reduce the likelihood of undiagnosed chronic systemic diseases, as no specific tests will be conducted to confirm their absence; participant responses will be accepted as valid. Additionally, requiring participants to already have a romantic partner ensures that the study is conducted ethically and avoids potential discomfort from pairing participants with strangers.<br>**Sociodemographic Characteristics:**<br>Participants must reside in **Guayaquil**, specifically near the **Universidad Católica de Santiago de Guayaquil**, to facilitate travel and ensure availability for multiple appointments.<br>**Sample Size:**<br>The study aims to recruit a minimum of **15 couples per group**, accounting for potential dropouts. This number was determined based on a sampling of means and a reference from a similar study.<br>**Selection Process:**<br>The recruitment process will involve an **open call** via social media platforms targeting university students, although being a student is not an inclusion criterion. Flyers with a QR code and a registration link will direct interested individuals to a **Google Form** where they will provide initial data. Eligible participants will be contacted to schedule an oral health assessment and complete a health questionnaire to confirm compliance with the inclusion criteria.
Interventions	11a	**Experimental Groups (Intervention):**<br>Participants in the experimental groups will engage in a **French kiss**, defined as a passionate kiss involving the exchange of salivary fluids through the mutual introduction of tongues into each other's mouths, along with lip contact. Participants will receive instructions to perform this kiss for a duration of **40 seconds**. While this duration is arbitrary and will be acknowledged as a limitation in the protocol, no prior studies provide a guideline for the specific duration of this type of kiss.<br>○ **Setting:** The intervention will take place in a private space within the dental clinic of the **Universidad Católica de Santiago de Guayaquil**. Each cubicle, equipped with a dental chair, will be enclosed with screens to ensure the privacy of participants. After completing the kiss, participants will leave the cubicle to provide an unstimulated saliva sample, which will be analyzed to measure pH.<br>**Control Group:**<br>Participants in the control group will not kiss but will still be required to bring their partners to ensure comparable conditions. The individual who consumes the acidic beverage will remain in the same cubicle for 40 minutes, providing unstimulated saliva samples every 5 minutes, as is required for the experimental groups. The control participants will remain seated and follow the same sample collection timeline as the intervention groups.<br>**Implementation Standards:**<br>○ The intervention (kissing) will be self-administered by participants. The research team will observe the initiation of the kiss to ensure it begins appropriately and will then start the timer

<table>
<tr>
<td></td>
<td></td>
<td>
for the 40-second duration. Once the timer concludes, participants will be asked to leave the cubicle and provide their saliva sample.

Tools and Equipment:

Screens (biombos) to provide privacy.
Timers or stopwatches to ensure accurate intervention durations and sample collection intervals.
A calibrated pH meter to measure salivary pH.
Eppendorf tubes (3ml) for saliva collection, suitable for accommodating the electrode of the pH meter and ensuring accurate measurements.

Monitoring and Compliance:
To respect participants' privacy, researchers will not remain present during the kiss. However, they will ensure the proper initiation of the intervention and verify compliance with the protocol. Saliva collection and pH measurement will be monitored closely to ensure adherence to the timeline.
Justification of the Intervention:
The kiss was chosen as the intervention due to its widespread and accessible nature. Demonstrating that kissing can act as a protective factor against the reduction of salivary pH—and potentially against dental caries—could contribute positively to preventive strategies for oral health. The study aims to explore the physiological impact of kissing in a controlled and measurable manner.
Materials and Equipment Used:

Eppendorf tubes (3ml): These will collect unstimulated saliva and accommodate the pH meter electrode, as only minimal saliva is expected per sample.
Calibrated pH meter: This device will measure salivary pH with precision.
Screens (biombos): To ensure the privacy and comfort of participants during the intervention.
Timers/Stopwatches: To accurately measure the duration of the kiss and sample collection intervals.

Safety and Monitoring Measures:
The intervention (kissing) is considered a safe and low-risk activity, especially as it will involve habitual partners. The clinic, located within the university, operates with official permits and adheres to national and international standards for disinfection and biosecurity. The privacy provided by screens further enhances participant comfort. In the unlikely event of an unforeseen incident, the university's medical department will be on standby to provide immediate assistance.
</td>
</tr>
<tr>
<td></td>
<td>11b</td>
<td>
Criteria for discontinuing or modifying allocated interventions for a given trial participant.
If any participant or couple feels uncomfortable or chooses not to continue with the kiss, they will be allowed to withdraw from the study without any consequences. In such cases, all data previously collected for their intervention will be excluded from the analysis.
</td>
</tr>
<tr>
<td></td>
<td>11c</td>
<td>
Strategies to improve adherence to intervention protocols, and any procedures for monitoring adherence.
To verify adherence, one of the investigators will observe the initiation of the kiss to ensure proper compliance and will start the timer for the specified 40 seconds. The investigator will notify the participants when the time is complete.
No further direct supervision will be performed during the kiss to respect the privacy of the participants.
</td>
</tr>
</table>

		The saliva samples collected after each intervention will be labeled and timestamped to ensure that the intervals between collections match the protocol (every 5 minutes).
	11d	**Relevant concomitant care and interventions that are permitted or prohibited during the trial.**<br>Participants will not be allowed to consume any food or drink, including water, for at least **two hours** before the start of the experiment. If any participant fails to adhere to this restriction, the couple will be rescheduled for another session.
Outcomes	12	**Primary Outcome:**<br>The primary outcome of this study is the **time required for salivary pH to return to neutral levels (pH ~7)** after the intervention (kissing). The objective is to demonstrate that the act of kissing facilitates a faster restoration of salivary pH compared to the physiological time required in the absence of a kiss.<br>**Secondary Outcomes:**<br>1. The **salivary pH level** measured at each time interval during the 40-minute observation period.<br>2. **Comparison of salivary pH restoration rates** between:<br>o The control group and the two experimental groups.<br>o Experimental group 1 (only one partner consumes the acidic beverage) and experimental group 2 (both partners consume the acidic beverage).<br>**Methods of Measurement:**<br>A pH meter will be used to measure the hydrogen ion activity in the saliva, reflecting its acidity or basicity. The device employs a **Toledo micro combined pH electrode** with the following specifications:<br>o **Measuring range:** pH 0 - 14.<br>o **Temperature range:** 0°C - 80°C.<br>o **Shaft dimensions:** 60mm length and 3mm diameter.<br>o **Glass membrane:** U-type with resistance <600M at 25°C.<br>o **Reference system:** ARGENTHAL with Ag+ ion trap.<br>The saliva sample will be **unstimulated** to avoid pH alterations caused by increased salivary flow. A small sample volume is expected, and the thin electrode design ensures accurate measurement even in reduced quantities.<br>**Measurement Timeline:**<br>1. A baseline saliva sample will be collected before the participants consume the acidic beverage.<br>2. Immediately after ingesting the beverage, a second saliva sample will be collected.<br>3. Participants in the experimental groups will kiss for 40 seconds, while the control group will wait.<br>4. A saliva sample will be collected at **5-minute intervals** for the next **40 minutes**, resulting in a total of nine post-intervention samples per participant.<br>**Justification:**<br>Measuring salivary pH every 5 minutes is a standard approach in similar studies assessing the dynamics of salivary pH restoration. Additionally, measuring pH immediately after consuming acidic beverages is consistent with other research protocols. Using a pH meter ensures precise and objective measurements, while collecting unstimulated saliva minimizes alterations in pH caused by increased flow rate, enhancing the reliability of the data.

Participant timeline	13	**See figure 1**
Sample size	14	The sample size calculation was conducted using the G*Power statistical software (version 3.1.9.7, Fran Faul, Universität Kiel, Germany). The calculation was based on data obtained from the pilot study titled "Comparison of Salivary pH Changes after Consumption of Two Sweetened Malaysian Local Drinks among Individuals with Low Caries Experience: A Pilot Study". This study measured salivary pH changes after consuming acidic beverages and provided the necessary statistical parameters for sample size estimation.<br>Specifically, the following values from the pilot study were used:<br>o Control group (mineral water): mean = 7.10, SD = 0.18.<br>o Experimental group (local drink): mean = 6.86, SD = 0.18.<br>To calculate the effect size (f) for ANOVA, the means and standard deviations were combined using the formula:<br>$$n = \frac{(2 * (Z\beta + Z\alpha)^2 * \sigma^2)}{\Delta^2}$$<br>Given the mean difference (7.10 - 6.86 = 0.24) and pooled SD of 0.18, d=1.33d = 1.33d=1.33. Dividing by the square root of the number of groups (k=3), the resulting effect size was f=0.76, which is considered a **large effect size**.<br>The following input parameters were used in G*Power:<br>o **Effect size (f):** 0.76 (large effect size based on observed data).<br>o **Alpha error probability (α):** 0.05.<br>o **Power (1-β):** 0.95.<br>o **Number of groups:** 3.<br>The output indicated that a total sample size of **30 participants** is required to achieve a power of 95%, with 10 participants per group. To account for potential dropouts, an additional 20% will be recruited, resulting in a total of **36 participants (12 per group).**<br>This effect size and resulting sample size are justified based on the observed differences in the pilot study, which measured salivary pH using similar methods (pH meter, unstimulated saliva samples in test tubes) and reported statistically significant differences between groups. Using this approach ensures that the sample size is both statistically adequate and logistically feasible for this study.
Recruitment	15	Participants will be recruited through an open call directed primarily at university students, although members of the general public who meet the inclusion criteria will also be eligible to participate. Recruitment announcements will be disseminated via social media platforms, university bulletin boards, and digital flyers, each containing a link or QR code to a Google Form where interested individuals can register.<br>The recruitment process will occur at the Universidad Católica de Santiago de Guayaquil, leveraging its student body and proximity to the study site. To ensure participants meet the eligibility criteria, an initial screening will be conducted using the information provided in the registration form. This screening will verify key inclusion and exclusion criteria, such as age range, oral health status, and the availability of a regular partner willing to participate.<br>To manage participation effectively, eligible participants will be scheduled for appointments at the university's dental clinic. Recruitment is expected to last two weeks, with a daily target of 5-10 pairs of participants screened

		and scheduled during this period. Participants will receive detailed information about the study during the informed consent process. No financial incentives will be offered; however, participants will be informed of the potential benefits of contributing to the advancement of dental health research.
**Methods: Assignment of interventions**		
Allocation: Sequence generation	16a	In this study, participants will be randomly assigned to one of three groups using the services of RANDOM.ORG, a reputable platform that generates true random numbers based on atmospheric noise .<br><br>Sequence Generation:<br>An external collaborator, Dr. Gabriela Zambrano from Miami, USA, will generate the allocation sequence. She will utilize the List Randomizer feature on RANDOM.ORG, which allows users to input a list of items (in this case, unique participant codes) and receive the list back in a randomized order. This method ensures that each participant has an equal chance of being assigned to any of the three groups. Random.org
Allocation concealment mechanism	16b	Allocation Concealment Mechanism:<br>To prevent bias, the allocation sequence will be concealed from both participants and primary investigators. Dr. Zambrano, who is not involved in the study and unaware of participant identities, will handle the randomization process. She will assign each unique participant code to a group and communicate these assignments confidentially to a designated coordinator. Participants will remain unaware of their group assignments to mitigate any anticipatory effects, such as increased salivation due to expectations.
Implementation	16c	Implementation:<br>During enrollment, each participant pair will receive a unique code. These codes will be sent to Dr. Zambrano, who will perform the randomization without access to any identifying information, ensuring impartiality. She will then relay the group assignments back to the study coordinator, who will oversee the implementation during the intervention phase. This process maintains the integrity of the randomization and minimizes potential biases.
Blinding (masking)	17a	Who Will Be Blinded:<br>This study will incorporate blinding at three levels to minimize bias and ensure the integrity of the results:<br>1. Participants: Participants will remain unaware of their group assignment (control or experimental) until the day of the experiment. This approach prevents anticipatory salivation or behavioral changes that could influence the results.<br>2. Investigators: The research team responsible for collecting and analyzing the data will not know the group assignments during the intervention phase. The assignments will only be revealed after data collection is complete to avoid any unintentional biases.<br>3. External Randomizer (Dr. Gabriela Zambrano): Dr. Zambrano, who performs the randomization process in Miami, USA, will be blinded to participant identities. She will only receive anonymized participant codes for allocation, ensuring impartiality in group assignments.
	17b	How Blinding Will Be Achieved:<br>o Participants: During recruitment and enrollment, all participants will be informed of the study procedures but will not be told whether they will be in the experimental or control groups. This

		information will only be disclosed on the day of the intervention to maintain blinding.<br>o Investigators: The assignment of participant codes to groups will be conducted by Dr. Zambrano using the RANDOM.ORG platform. The research team will only receive group assignments linked to participant codes after all data has been collected.<br>o External Randomizer: Dr. Zambrano will not have access to any identifying information about participants. She will receive only the anonymized codes, ensuring she remains blinded to the participants' identities.
**Methods: Data collection, management, and analysis**		
Data collection methods	18a	The primary outcome of this study is the time required for salivary pH to return to neutral (pH 7.0) after the kissing intervention, compared to the control group. Baseline data, including the initial pH of unstimulated saliva before the intervention, will also be collected. Secondary outcomes include salivary pH measurements at defined time intervals (e.g., immediately after the intervention and every 5 minutes up to 40 minutes).<br>Data Collection Process:<br>o Participants will expectorate saliva into sterile 3 mL conical Eppendorf tubes at each designated time point.<br>o A calibrated pH meter will be used to measure the pH of the collected saliva. Calibration will be performed using standard buffer solutions at pH 4.01 and pH 7.01 before each data collection session, ensuring accuracy and reliability of the measurements.<br>o All pH measurements will be conducted by trained members of the research team, who will follow a standardized protocol to minimize variability and ensure data quality. Duplicate measurements will be taken for 10% of the samples to verify consistency.<br>Data Recording:<br>o Measured values will be entered directly into a secure, cloud-based Excel spreadsheet accessible only to authorized members of the research team. This ensures data integrity and confidentiality.
	18b	To ensure participant retention and maximize data completeness, the following strategies will be implemented:<br>1. **Clear Communication:** Participants will receive detailed explanations of the study procedures and expectations during the informed consent process. This includes emphasizing the importance of completing all saliva sample collections.<br>2. **Convenient Scheduling:** Appointments will be scheduled at times that are convenient for participants to reduce the likelihood of dropouts.<br>3. **Supportive Environment:** Participants will have access to a comfortable waiting area during the study to ensure a positive experience.<br>**Handling Dropouts:**<br>o If a participant chooses to withdraw from the study, all data collected from that individual will be permanently deleted from the database. This policy ensures respect for the participant's decision and aligns with the principles of informed consent.<br>o A log will be maintained to record cases of withdrawal, including the number of participants who drop out and their reasons (if voluntarily disclosed), but no data from these participants will be included in the final analysis.

Data management	19	All data collected during the study will be securely managed and protected to ensure participant confidentiality and data integrity. The following procedures will be implemented:<br>1. Data Collection and Recording:<br>o Salivary pH measurements and participant identifiers (coded) will be recorded directly in a secure online Excel spreadsheet hosted on a password-protected cloud platform (e.g., Google Drive).<br>o Access to the spreadsheet will be restricted to the research team through individual accounts with two-factor authentication.<br>2. Data Anonymization:<br>o Participants will be assigned unique alphanumeric codes during the enrollment process. All data will be stored and analyzed using these codes, ensuring that no identifying information is linked to the results.<br>o The key linking participant names to their codes will be stored separately in a secure location and will only be accessible to the principal investigator.<br>3. Data Storage and Security:<br>o All electronic data will be stored in a cloud-based system with encrypted storage and access control.<br>o Physical documents, such as consent forms, will be stored in a locked cabinet within the principal investigator's office.<br>4. Access and Use:<br>o Only authorized members of the research team will have access to the data. Each member will sign a confidentiality agreement prior to accessing any study-related information.<br>5. Data Retention and Deletion:<br>o Study data will be retained for a period of five years following the publication of the results, in accordance with institutional and international guidelines.<br>o After the retention period, all electronic data will be permanently deleted, and physical documents will be shredded.
Statistical methods	20a	Analysis of Primary and Secondary Outcomes:<br>The primary outcome, the time required for salivary pH to return to neutral (pH 7.0), will be analyzed using appropriate statistical methods depending on the data distribution. First, tests of normality (e.g., Shapiro-Wilk test) will be conducted to determine if the data follows a normal distribution. If the data meets the assumptions for parametric tests, a one-way ANOVA will be used to compare the mean recovery times across the three groups: control, experimental group 1 (only one partner drinks the acidic beverage), and experimental group 2 (both partners drink the acidic beverage).<br>If the assumptions for ANOVA are not met, the non-parametric Kruskal-Wallis test will be applied to compare the groups. Post-hoc analyses (e.g., Tukey's HSD for ANOVA or pairwise comparisons with Bonferroni correction for Kruskal-Wallis) will be conducted to identify specific group differences.<br>For secondary outcomes, including salivary pH levels at specific time intervals, repeated-measures ANOVA will be used if the data meets parametric assumptions. Otherwise, the Friedman test will be employed to evaluate changes in pH over time within and between groups.<br>Additionally, pairwise comparisons will be performed to assess the differences in mean recovery times between the control group and each experimental group (control vs. experimental 1, and control vs.

		experimental 2). These comparisons will utilize independent t-tests if parametric assumptions are met or Mann-Whitney U tests if they are not.
	20b	Missing data will be managed using the following approach:<br>1. Complete-Case Analysis: Only participants with complete data for the primary outcome will be included in the primary analysis.<br>2. Sensitivity Analysis: If more than 10% of the data are missing, sensitivity analyses will be conducted using multiple imputation methods to assess the robustness of the findings.<br>3. Documentation of Missing Data: All instances of missing data will be documented, and the reasons for missingness (if known) will be recorded in a study log.
**Methods: Monitoring**		
Data monitoring	21a	Composition of Data Monitoring Committee (DMC): This study does not include a formal Data Monitoring Committee (DMC) due to its low-risk nature and straightforward methodology. The intervention (kissing) is non-invasive, and no adverse effects are anticipated. Data monitoring responsibilities will be undertaken by the principal investigator (PI) and the research team, who will oversee data collection, ensure adherence to the protocol, and verify data quality.
	21b	**Interim Analyses and Stopping Guidelines:** No interim analyses are planned for this study, as the duration and design do not necessitate ongoing assessments of outcome data. The study will continue as planned unless unforeseen ethical concerns or logistical issues arise. The principal investigator will have the authority to decide on early termination in consultation with the research ethics committee if necessary.
Harms	22	**Plans for Monitoring and Managing Adverse Events:**<br>This study involves a low-risk intervention (kissing) with no anticipated adverse events or unintended effects. However, to ensure participant safety and address any unexpected occurrences, the following procedures will be implemented:<br>1. Identification and Collection of Adverse Events:<br>o Participants will be instructed to report any discomfort, adverse reactions, or unintended effects immediately to the research team, either verbally or via a follow-up questionnaire administered at the end of the study session.<br>o Potential adverse events that may be monitored include emotional discomfort, physical reactions related to saliva exchange, or unforeseen logistical issues during the study procedures.<br>2. Assessment of Adverse Events:<br>o Any reported adverse event will be documented and evaluated by the principal investigator to determine its severity (mild, moderate, severe) and its relation to the study intervention.<br>3. Reporting of Adverse Events:<br>o All adverse events will be recorded in a study log and reported to the institutional ethics committee if they are deemed related to the study intervention or conduct.<br>o Serious adverse events will be reported within 24 hours to the ethics committee and the relevant regulatory bodies, if applicable.<br>4. Management of Adverse Events:<br>o Participants experiencing discomfort or adverse effects will be provided with appropriate support, including medical attention if necessary.<br>o In the unlikely event of a serious adverse effect, the study may be temporarily paused or terminated after consultation with the ethics committee.

		These measures ensure that any unintended effects of the study are addressed promptly and effectively, maintaining participant safety and ethical standards.
Auditing	23	**Formal audits are not planned for this study due to its low-risk nature and straightforward design. However, the following internal monitoring procedures will be implemented to ensure compliance with the study protocol and data quality:**<br>1. Internal Monitoring by the Principal Investigator (PI):<br>o The PI will conduct bi-weekly reviews of study activities, including data collection processes, adherence to the protocol, and participant consent documentation.<br>2. Documentation and Reporting:<br>o All monitoring activities, including identified issues and corrective actions, will be recorded in a monitoring log. This log will be available for review by the institutional ethics committee upon request.<br>3. Independent Oversight:<br>o While no formal independent audits are planned, the study remains subject to oversight by the institutional ethics committee, which may conduct random reviews or request updates at any time.<br>Given the nature of the study, these internal monitoring practices are deemed sufficient to ensure the integrity of the trial and compliance with ethical standards.
**Ethics and dissemination**		
Research ethics approval	24	The study received ethical approval from the Ethics Committee of the Instituto Superior Universitario "Portoviejo" under the project title: "Potencial efecto protector del beso bucal entre dos personas contra la disminución del pH salival". The approval number is 1681428096.<br>**1. Institutional Compliance:**<br>The Ethics Committee of the Instituto Superior Universitario "Portoviejo" is approved by the Ministry of Public Health of Ecuador, which oversees and authorizes all committees permitted to approve research involving human subjects within the country.<br>**2. Ethical Standards:**<br>This study adheres to the ethical principles outlined in the Declaration of Helsinki and complies with local regulations and international guidelines for Good Clinical Practice (GCP). Participant confidentiality, informed consent, and the right to withdraw without penalty are strictly upheld throughout the study.<br>**3. Documentation and Record Keeping:**<br>A copy of the ethics approval letter will be maintained for the duration of the study and will be made available for audits or for submission to journals as required for publication.
Protocol amendments	25	The study protocol has been registered in ClinicalTrials.gov under the identifier NCT06501729. Any important protocol modifications, such as changes to eligibility criteria, outcomes, or statistical analyses, will be communicated promptly to all relevant parties as outlined below:<br>**1. Communication with Ethics Committees:**<br>o **A**ll protocol amendments will be submitted for review and approval to the Ethics Committee of the Instituto Superior Universitario "Portoviejo", following the same process used for the initial protocol approval.<br>o The ethics committee will be provided with detailed documentation of the proposed changes, including justifications

		and any potential implications for participant safety or study validity.<br>**2. Notification to Investigators and Research Team:**<br>o The principal investigator will immediately inform all members of the research team about any approved modifications. A meeting or written communication will be used to ensure that all team members understand and implement the changes consistently.<br>**3. Updates to ClinicalTrials.gov:**<br>o Protocol modifications will be updated in the ClinicalTrials.gov registry under NCT06501729. This update will include a clear description of the changes and the date they were implemented.<br>**4. Communication with Participants:**<br>o If the modifications directly affect participants (e.g., changes to eligibility criteria or interventions), they will be informed through written communication and updated consent forms. Participants will have the opportunity to ask questions and, if necessary, withdraw from the study without any consequences.<br>**5. Journals, Regulators, and Other Stakeholders:**<br>o Protocol changes that affect the study's primary outcomes or methodology will be disclosed to journals or regulatory bodies involved in the trial's dissemination or oversight. This ensures transparency and compliance with publication standards.<br>**6. Documentation:**<br>o All protocol amendments, communications, and approvals will be documented and stored in the study records for audit purposes.
Consent or assent	26a	**Obtaining Informed Consent:**<br>**Informed consent will be obtained from all participants prior to their inclusion in the study. The process will involve the following steps:**<br>1. A trained member of the research team will explain the study objectives, procedures, potential risks, and benefits in clear and simple language.<br>2. Participants will be provided with a copy of the informed consent form, which has been approved by the Ethics Committee of the Instituto Superior Universitario "Portoviejo" (CEISH-ITSUP). This form includes comprehensive details about the study and outlines participants' rights, including the option to withdraw at any time without penalties.<br>3. Participants will be given sufficient time to review the form and ask questions. Both members of each couple must provide their consent to participate in the study.<br>4. The investigator responsible will document the informed consent process, including the participant's signature and the date of consent.
	26b	**Additional Consent Provisions:**<br>**The informed consent form includes explicit provisions for the collection and use of participants' personal data and biological specimens (unstimulated saliva). These provisions ensure:**<br>• Data Privacy: All data will be anonymized using a coding system, preventing identification of participants in published results or during analysis.<br>• Specimen Use: Saliva samples will be used exclusively for pH measurement as part of this study. No samples will be stored or reused for other research purposes.

		• Withdrawal Rights: Participants may request the deletion of their data and the destruction of their samples at any time during the study.<br>The approved informed consent form explicitly addresses these provisions, guaranteeing participants' autonomy and adherence to ethical standards.
Confidentiality	27	**Plans for Protecting Participant Confidentiality**<br>**The study ensures the protection of personal information for all potential and enrolled participants through the following measures:**<br>1. Collection of Personal Information:<br>o Personal information, including participants' names, contact details, and unique identification codes, will be collected during enrollment and stored separately from study data.<br>o All personal identifiers will be replaced with alphanumeric codes in the study database to maintain anonymity.<br>2. Data Sharing and Access:<br>o Only authorized members of the research team will have access to personal information and coded data. Access will require two-factor authentication for digital records and signed confidentiality agreements for team members.<br>o No personal information will be shared with third parties, journals, or external stakeholders.<br>3. Data Maintenance and Security:<br>o Electronic data will be stored on a secure cloud-based system with encrypted storage and password protection.<br>o Physical documents, such as consent forms, will be stored in a locked cabinet within the principal investigator's office.<br>o A master list linking participant codes to personal information will be maintained in a secure file accessible only to the principal investigator.<br>4. Confidentiality During and After the Trial:<br>o During the trial, all results and data analysis will use anonymized codes to prevent identification of participants.<br>o After the trial, all personal information will be permanently deleted five years after the publication of study results, in accordance with institutional and regulatory guidelines. Anonymized data may be retained for secondary analysis or publication purposes, without any link to personal identifiers.
Declaration of interests	28	Declaration of Financial and Competing Interests<br>The study is funded by the Universidad Católica de Santiago de Guayaquil, which provides the necessary resources, equipment, and materials for its execution. The funding is strictly institutional, with no external financial contributions or sponsorships. All authors are employees or affiliates of this institution, and their involvement in the study is independent of any financial or organizational incentives.<br>Specifically:<br>**1. Funding Source:**<br>o The Universidad Católica de Santiago de Guayaquil fully supports this study through institutional funds, ensuring the research is conducted without external financial influence.<br>**2. Financial Independence of Investigators:**<br>o The authors have no personal financial investment or sponsorship that could bias the study design, execution, or reporting.

		o None of the investigators receive financial compensation beyond their institutional roles related to this research.<br>3. **Competing Interests:**<br>o The external collaborator responsible for randomization, Dr. Gabriela Zambrano, is a professor affiliated with the Universidad Católica de Santiago de Guayaquil and provides voluntary support to this study. While she is associated with the institution, her role is limited to conducting the randomization process remotely from Miami, USA, ensuring she remains independent from other aspects of the study.<br>o No other professional or personal affiliations could influence the objectivity of the investigators.
Access to data	29	**Access to Data**<br>Access to the full dataset will be restricted to the principal investigator and the designated members of the research team. This ensures the confidentiality of participant information and maintains the integrity of the data throughout the study.<br>**1. Data Access Policy:**<br>o Only authorized members of the research team will have access to the complete dataset during the study.<br>o Access will require authentication via secure login credentials to the cloud-based storage system.<br>o Each team member will sign a confidentiality agreement outlining their responsibilities for data handling and protection.<br>**2. Conditions for External Access:**<br>o Requests for access to anonymized data for secondary analysis or replication studies will be considered only after the study's primary results have been published.<br>o External access will require approval from the principal investigator and the Ethics Committee of the Instituto Superior Universitario "Portoviejo."<br>o All shared data will be anonymized to ensure that no identifying information about participants is disclosed.<br>**3. Long-Term Data Access:**<br>o An anonymized version of the dataset will be archived securely for five years after the study's completion, in compliance with institutional and international research guidelines.<br>o After this period, the dataset will be permanently deleted unless additional ethical approvals are obtained for its continued use.
Ancillary and post-trial care	30	**Ancillary and Post-Trial Care**<br>**Given the non-invasive and low-risk nature of the intervention (kissing), no ancillary or post-trial care is anticipated to be necessary for participants. However, the following provisions are in place to address any unexpected concerns:**<br>1. **Post-Trial Contact Information:** Participants will receive the contact details of the principal investigator, who will be available to address any questions or concerns following their participation in the study.<br>2. **Access to Medical Care:** Should any participant experience discomfort or a perceived adverse effect related to the study, they will have access to the medical facilities of the Universidad Católica de Santiago de Guayaquil, where the study is conducted. The university's medical staff will provide free consultation and basic treatment if necessary.

		3. **Post-Trial Feedback:** Participants will have the opportunity to request a summary of the study's results once the analysis is complete. This ensures transparency and acknowledges their contribution to the research.
Dissemination policy	31a	**Plans for Disseminating Study Results**<br>o The findings of this study will be disseminated through publication in a peer-reviewed journal of high impact in the fields of dentistry and public health.<br>o Results will also be presented at national and international conferences to share insights with the broader scientific community.
	31b	**Authorship Eligibility Guidelines and Any Intended Use of Professional Writers**<br>o Authorship will be determined based on the guidelines of the International Committee of Medical Journal Editors (ICMJE), ensuring that all contributors who meet the criteria for authorship are included.<br>o No professional writers will be used in the preparation of manuscripts. The research team, led by the principal investigator, will take full responsibility for drafting and revising all study-related publications.
	31c	**Plans, if Any, for Granting Public Access to the Full Protocol, Participant-Level Dataset, and Statistical Code**<br>o An anonymized version of the dataset will be made publicly available in an appropriate research data repository after publication, in line with open science principles and journal requirements.<br>o Results and updates will also be added to the ClinicalTrials.gov registry under the identifier NCT06501729 to ensure transparency and accessibility.<br>o The full protocol and statistical code will be shared upon reasonable request to the principal investigator, subject to approval by the institutional ethics committee.
Informed consent materials	32	**Appendices 1**
Biological specimens	33	**Appendices 2**

Figure 1. Schedule of Enrolment, Interventions, and Assessments

**Study Period**	**Enrolment**	**Allocation**	**Post-Allocation**	**Close-Out**
**Timepoint**	Week 1-2	Week 3	Week 3-10	Week 11-12
**Enrolment:**				
- Eligibility screening	X			
- Informed consent	X			
- Baseline assessments		X		
**Interventions:**				
- Group Experimental 1			X	
- Group Experimental 2			X	
- Group Control			X	
**Assessments:**				
- Immediate post-intervention			X	
- Follow-up (every 5 min up to 40 min)			X	
**Data Analysis:**				X

Appendices 1. Informed Consent

Thank You for Your Interest:
Thank you for accessing the link that brought you here. Your participation in this research is invaluable to advancing scientific knowledge.

Study Title:
"Potential Protective Effect of Kissing on Salivary pH Recovery After Acidic Beverage Consumption"

Research Team:
This study is led by Dr. Marcelo Armijos Briones, faculty researcher from the Dentistry Program at Universidad Católica de Santiago de Guayaquil (UCSG), with a team of experienced researchers.

Objective of the Study:
We aim to investigate whether the act of kissing can help restore salivary pH after consuming three types of beverages: Cifrut, Coca-Cola, and non-alcoholic beer.

Procedures:
If you decide to participate, the following steps will be conducted:

1. **Initial Interview:** We will assess your general health status using a questionnaire.
2. **Oral Examination:** We will evaluate your oral health to ensure you meet the study criteria. If you have significant dental caries or any condition that affects salivary pH, you will not be eligible.
3. **Saliva Collection:** You will provide six saliva samples at different time points during the experiment.
4. **Beverage Consumption:** You will consume one of the mentioned beverages to assess its effect on your salivary pH. If you are allergic or intolerant to these beverages, you will not be able to participate.
5. **Experimental Intervention:** If assigned to the experimental group, you will kiss your partner for 2 minutes. If not, no kissing will be required.
6. **Study Duration:** You will attend four sessions, each focusing on the effect of a different beverage and intervention.

Privacy and Confidentiality:
The saliva collection and kissing process will take place in a private setting within the UCSG dental clinics. Your personal information will be treated with strict confidentiality and protected according to national laws and international ethical standards.

Voluntariness and Rights:
Participation is entirely voluntary. You can withdraw at any time without justification and without affecting your relationship with the university or the research team. If you withdraw, all collected data will be permanently deleted.

Risks and Benefits:
No significant risks are anticipated. While there is no monetary compensation, your contribution will be invaluable to advancing scientific research.

Ethical Approval:
This study has been approved by the Ethics Committee of the Instituto Superior Universitario "Portoviejo" (ITSUP), approval number 1681428096.

Contact Information:
For questions or further information, you can contact the principal investigator:
Dr. Marcelo Armijos Briones
Email: fernando.armijos@cu.ucsg.edu.ec
Phone: 0969711767

Consent:
By signing this document, you agree to participate in the study under the explained terms.

Participant Signature:
Name: __________________________
Date: ____________________________
Signature: ______________________

Appendices 2. Plan for Handling Biological Specimens

Below is a detailed plan for handling biological specimens (saliva) collected during the study. This document is prepared to comply with institutional and international ethical guidelines and can be included as an appendix to the protocol.

Plan for Handling Biological Specimens: Saliva Samples

1. Collection of Samples:

- Saliva samples will be collected from participants using **sterile, 3 mL conical Eppendorf tubes**.
- Samples will be collected at predefined time intervals:
  - Before consuming the assigned beverage (baseline).
  - Immediately after consuming the beverage.
  - At regular intervals following the intervention (e.g., every 5 minutes).
- Unstimulated saliva will be collected to avoid altering the natural pH.

2. Immediate Analysis:

- Saliva samples will be analyzed immediately after collection using a calibrated **pH meter**.
- Calibration will be performed prior to each session with standard buffer solutions at pH 4.01 and pH 7.01 to ensure accuracy.

3. Anonymization and Labeling:

- Each sample will be labeled with a unique alphanumeric code that corresponds to the participant's anonymized identifier.
- No personal identifiers (e.g., names or contact information) will be included on the sample labels.

4. Storage and Disposal of Samples:

- Samples will not be stored for future research.
- After the pH measurements are completed, the samples will be disposed of immediately in accordance with the **biosafety regulations of Universidad Católica de Santiago de Guayaquil**.
- Disposal will follow local and national guidelines for biological waste management, ensuring that samples are handled responsibly and ethically.

5. Purpose of Use:

- Saliva samples will be used exclusively for measuring pH as part of the current study.
- There are no plans to conduct ancillary analyses or additional research using these samples.

6. Compliance with Ethical Standards:

- This study adheres to the ethical principles outlined in the **Declaration of Helsinki** and complies with institutional and national guidelines for the handling of biological specimens.
- The process ensures participant confidentiality and the ethical use of biological materials.

Signature and Approval

Prepared by:
Dr. Marcelo Armijos Briones
Principal Investigator
